# CORPORATE PERSPECTIVES OF A NATIONAL NURSING HOME PRAGMATIC TRIAL AND FACTORS AFFECTING IMPLEMENTATION FIDELITY

**DOI:** 10.1093/geroni/igad104.1543

**Published:** 2023-12-21

**Authors:** Jacy Weems, Ellen McCreedy, Grace Wittenberg, Rosa Baier

**Affiliations:** Brown University School of Public Health, Providence, Rhode Island, United States; Brown University School of Public Health, Providence, Rhode Island, United States; Brown University School of Public Health, Providence, Rhode Island, United States; Brown University School of Public Health, Providence, Rhode Island, United States

## Abstract

Implementation fidelity is concerning for embedded pragmatic clinical trials (ePCTs), where effectiveness is evaluated in real-world settings instead of a structured clinical environment. Healthcare facilities paused ePCT research activities during the COVID-19 pandemic to address related staffing issues and infections. We examined a 54-nursing-home ePCT implementing Music & Memory, a nonpharmacological intervention using music to improve quality of life for residents living with dementia. Our qualitative study describes factors influencing implementation fidelity across four nursing home corporations. The interview guide assesses corporate perceptions of implementation barriers, facilitators, and participation incentives. Themes were organized within the Conceptual Framework for Implementation Fidelity’s potential modifying factors: 1) context, 2) participant responsiveness, 3) intervention complexity, 4) facilitation strategies, 5) quality of delivery, and 6) recruitment. Interviewees (n=4) lead corporate-wide dementia initiatives. 1) Context: COVID-19-related capacity issues and local “champion” turnover impacted success. 2) Participant responsiveness: Developing playlists strengthened staff-resident relationships, although time-consuming and requiring staff competency. Residents experienced decreased agitation while staff satisfaction increased. 3) Intervention complexity: Brown’s research team adapted processes to save staff time. 4) Facilitation strategies: The relationship with Brown promoted problem-solving. 5) Quality of delivery: The ePCT was an effective, non-pharmacological approach addressing dementia symptoms. 6) Recruitment: Corporate leadership makes decisions regarding individual nursing homes’ recruitment in research. Financial incentives did not drive participation, although some voiced appreciation for offsetting time and resources invested. Recommendations for future efforts included in-person champion training and site-specific quality improvement efforts. Researchers maintaining relationships with corporate and local leadership promotes sustainable engagement.

